# Elucidation of the mechanism of action of ailanthone in the treatment of colorectal cancer: integration of network pharmacology, bioinformatics analysis and experimental validation

**DOI:** 10.3389/fphar.2024.1355644

**Published:** 2024-02-07

**Authors:** Shanbo Ma, Xiaodi Guo, Ruisi Han, Qian Meng, Yan Zhang, Wei Quan, Shan Miao, Zhao Yang, Xiaopeng Shi, Siwang Wang

**Affiliations:** ^1^ The College of Life Science, Northwest University, Xi’an, Shaanxi, China; ^2^ Department of Pharmacy, Affiliated Hospital of Shaanxi University of Chinese Medicine, Xianyang, Shaanxi, China; ^3^ Department of Pharmacy, Xijing Hospital, Air Force Medical University, Xi’an, Shaanxi, China; ^4^ Department of Military Medical Psychology, Air Force Military Medical University, Xi’an, Shaanxi, China

**Keywords:** ailanthone, colorectal cancer, network pharmacology, bioinformatic, PI3K/AKT

## Abstract

**Background:** Ailanthone, a small compound derived from the bark of *Ailanthus altissima* (Mill.) Swingle, has several anti-tumour properties. However, the activity and mechanism of ailanthone in colorectal cancer (CRC) remain to be investigated. This study aims to comprehensively investigate the mechanism of ailanthone in the treatment of CRC by employing a combination of network pharmacology, bioinformatics analysis, and molecular biological technique.

**Methods:** The druggability of ailanthone was examined, and its targets were identified using relevant databases. The RNA sequencing data of individuals with CRC obtained from the Cancer Genome Atlas (TCGA) database were analyzed. Utilizing the R programming language, an in-depth investigation of differentially expressed genes was carried out, and the potential target of ailanthone for anti-CRC was found. Through the integration of protein-protein interaction (PPI) network analysis, GO and KEGG enrichment studies to search for the key pathway of the action of Ailanthone. Then, by employing molecular docking verification, flow cytometry, Transwell assays, and Immunofluorescence to corroborate these discoveries.

**Results:** Data regarding pharmacokinetic parameters and 137 target genes for ailanthone were obtained. Leveraging The Cancer Genome Atlas database, information regarding 2,551 differentially expressed genes was extracted. Subsequent analyses, encompassing protein–protein interaction network analysis, survival analysis, functional enrichment analysis, and molecular docking verification, revealed the PI3K/AKT signaling pathway as pivotal mediators of ailanthone against CRC. Additionally, the *in vitro* experiments indicated that ailanthone substantially affects the cell cycle, induces apoptosis in CRC cells (HCT116 and SW620 cells), and impedes the migration and invasion capabilities of these cells. Immunofluorescence staining showed that ailanthone significantly inhibited the phosphorylation of AKT protein and suppressed the activation of the PI3K/AKT signaling pathway, thereby inhibiting the proliferation and metastasis of CRC cells.

**Conclusion:** Therefore, our findings indicate that Ailanthone exerts anti-CRC effects primarily by inhibiting the activation of the PI3K/AKT pathway. Additionally, we propose that Ailanthone holds potential as a therapeutic agent for the treatment of human CRC.

## 1 Introduction

Colorectal cancer (CRC) is among the most prevalent gastrointestinal malignancies (Liu et al., 2022). According to the World Cancer Progress Report 2020 by the World Health Organization, CRC is the third and second most common malignancy in terms of incidence and mortality, respectively, among all cancer types. Importantly, it has a higher incidence in men compared with women (Sung et al., 2021). In China, the occurrence and fatality rates of CRC show an upward trend, with it having the second-highest incidence rate and fourth-highest mortality rate among malignant tumors (Xia C. et al., 2022a; Zheng et al., 2022). Despite major advances in diagnostic techniques and treatment strategies, the prognosis of patients with CRC remains poor, with over half of those with advanced disease eventually dying because of metastasis and recurrence (Siegel et al., 2023; Zhang et al., 2023). Currently, surgery, chemotherapy, radiotherapy, and biotherapy are commonly employed for CRC treatment. However, they pose challenges such as low target specificity, toxic side effects, heavy economic burden, and poor patient compliance, often leading to treatment discontinuation or poor efficacy (Yaghoubi et al., 2020; Wan et al., 2022). Active ingredients in traditional Chinese medicine offer early treatment responses, symptom improvement, prolonged survival, immune function regulation, and enhanced quality of life for patients with CRC (Guo et al., 2012; Li et al., 2021b).

Ailanthone (AIL, C_20_H_24_O_7_, Supplementary Figure S1), derived from the dried bark of *Ailanthus altissima* (Mill.) Swingle, holds a significant place in traditional Chinese medicine (De Feo et al., 2003). AIL is a pentacyclic diterpene lactone and is renowned for its versatile medicinal properties, including antiviral (Kubota et al., 1997), antitubercular (Rahman et al., 1997), antimalarial (Okunade et al., 2003), anti-inflammatory (Li X. et al., 2021a), and antitumor effects (Chen et al., 2017; Daga et al., 2019). Numerous studies have highlighted its potent antitumor capabilities, demonstrating efficacy in prostate cancer through the marked downregulation of RPA1, a key player in DNA replication (He et al., 2016). In addition, AIL exhibits promise in treating nonsmall cell lung and liver cancers (Zhuo et al., 2015; Ni et al., 2017). Although extensively studied in various malignancies, such as melanoma (Liu et al., 2019), acute myelogenous leukemia (Wei et al., 2018), bladder cancer (Daga et al., 2019), breast cancer (Wang et al., 2018), gastric cancer (Chen et al., 2017), and vestibular neuroma (Yang et al., 2018), its role in CRC remains poorly understood.

Network pharmacology is employed to identify candidate targets and elucidate bioactive compound functions in disease treatment and bioinformatics analysis is instrumental in investigating complicated gene–disease relationships and regulatory mechanisms (Wang et al., 2022). These approaches have deepened our understanding regarding the action mechanisms of drugs (Zou et al., 2022), successfully revealing the multitargeted pharmacological roles of several compounds including *Fumaria indica* and the Phellodendron–Anemarrhena drug pair in hepatocellular carcinoma treatment (Batool et al., 2022; Ruan et al., 2022). Similarly, Xia et al. used bioinformatics and network pharmacology to determine the inhibition mechanism of luteolin on the proliferation and migration of glioblastoma cells through the key targets BIRC5 and CCNB1, which impacted the prognosis of patients with glioblastoma (Xia Z. et al., 2022b). This interdisciplinary approach synergizes data mining and integration, enabling disease mapping to potential therapeutic agents.

This study investigated the complex mechanisms, pathways and survival outcomes of AIL in the treatment of CRC. We used a combination of network pharmacology and bioinformatics techniques to uncover the therapeutic potential of AIL. Cellular assays were performed to substantiate these findings, providing a comprehensive exploration of the role of AIL in the treatment of CRC.

## 2 Materials and methods

### 2.1 Evaluation of ADME for AIL

Predictive analysis of the drug-forming potential of AIL using the SwissADME database (http://www.swissadme.ch/) encompassed six parameters; physiochemical properties, lipid solubility, water solubility, pharmacokinetic properties, drug-like properties, and medicinal properties (Huang et al., 2022).

### 2.2 Target identification for AIL

A comprehensive search for AIL target genes using TCMSP, HERB, Swiss Target Prediction, and PharmMapper databases was conducted. The keyword “ailanthone” served as the query for this search to screen relevant AIL targets. To ensure consistency and standardize target gene nomenclature (gene symbols), the UniProt database (https://sparql.uniprot.org/) (Han et al., 2023) was employed.

### 2.3 Data collection and annotation

Acquisition of RNA sequencing data from 487 samples from the TCGA database was followed by the annotation of these data using the Perl programming language to convert the probe matrix into a gene matrix.

### 2.4 Differentially expressed gene (DEG) identification

To identify genes with distinct expression patterns between the tumor and normal groups, a transcriptomic analysis was executed using the “LIMMA” package in the R programming language. The filter criteria of |LogFC| ≥ 1 and corrected *p* < 0.05 were used. Volcano plots and heatmaps were generated to visualize the DEGs using the “pheatmap” package in R language.

### 2.5 Mapping disease and drug gene interactions

Target genes associated with AIL were intersected with DEGs observed in CRC. The “VennDiagram” package in R was employed to create Venn diagrams, which illustrated the intersecting genes between the two datasets.

### 2.6 PPI network construction

For PPI network construction, the intersecting genes between AIL and CRC were identified. Network topology analysis was then conducted using the online platform STRING-DB (https://string-db.org/). The resulting “tsv” file was downloaded and imported into Cytoscape 7.2.1 using the “cytomubba” plugin for core gene screening. A PPI network diagram featuring central genes was generated. Additionally, the “string_interactions.tsv” file was used in R to create a histogram illustrating core gene interactions.

### 2.7 Verification of core genes

The “survival” and “survminer” packages in R (Liu et al., 2021) were used to perform an analysis of the variation in expression levels of these 10 core genes between normal and CRC tissues in a total of 446 CRC samples. Furthermore, in order to further analyse the prognostic significance of these core genes, we employed the Kaplan-Meier online platform (Song et al., 2023) to assess the impact of these core genes on overall survival in 1061 CRC samples.

### 2.8 Functional enrichment analysis

To gain insights into the biological significance of the identified genes, functional enrichment analyses were performed incorporating GO terms (http://GeneOntology.org/) and KEGG pathways (https://www.kegg.jp/eg/) (Yu et al., 2023). R packages (Yu et al., 2023), including “clusterProfiler,” “org.Hs.eg.db,” “enrichplot,” “ggplot2,” and “DOSE,” were used with specified filter conditions: “*p*-value cutoff = 0.05” and “q value cutoff = 0.05.” GO enrichment analysis encompassing CC, BP, and MF outcomes were presented in a visually informative bubble chart. Similarly, the KEGG pathway enrichment analysis outcomes were depicted in a column chart.

### 2.9 Construction of the “AIL-CRC-intersecting Gene-KEGG” regulatory network

Cytoscape7.2.1 was used to visualize the “AIL–CRC–intersection gene–KEGG” network and elucidate the AIL–CRC relationship as well as its corresponding pathways.

### 2.10 Validation of molecular docking between AIL and targets

To further explore the key targets of the anti-CRC effect of AIL, molecular docking analysis was performed between AIL and potential core targets identified in the PPI analysis and key proteins PI3K and AKT in the PI3K/AKT signaling pathway screened by KEGG enrichment analysis. The molecular docking process involved several sequential steps (Trott and Olson, 2010; Eberhardt et al., 2021):

1). Acquisition of AIL Structure:
- The SDF structure diagram of AIL was downloaded from PubChem.
- Subsequently, the structure was imported into Chem3D software to create a 3D structure diagram of the core compound.

- The energy of the 3D structure was optimized, and the resulting structure was saved in the mol2 format.

- Finally, this optimized structure was imported into AutoDockTools-1.5.6 software and saved in the pdbqt format.
2). Acquisition of Protein Structures:
- Crystal structure diagrams of the proteins from the targets set were retrieved from the PDB and were downloaded.
- These protein structures were further imported into Pymol software to eliminate water molecules and small molecules.
- Using AutoDockTools-1.5.6 software, hydrogen atoms were added and necessary charge operations were performed. The file was saved in the pdbqt format.
3). Molecular Docking:
- The docking pocket locations were determined.
- Auto Dock Vina was used to conduct molecular docking simulations.

### 2.11 Cell culture

CRC cells (HCT116 and SW620 cells obtained from the Chinese Academy of Sciences, Beijing, China) were cultured in RPMI-1640 medium containing 10% fetal bovine serum and 1% penicillin–streptomycin. They were incubated at 37°C in a 5% CO_2_ environment. When cellular density reached 80%–90%, trypsin (0.25%) was used for cell digestion and passaging.

### 2.12 MTT assay for cell proliferation

CRC cells (SW620 and HCT116) in their logarithmic growth phase were seeded into 96-well culture plates at a density of 1 × 10^4^ cells per well (100 μL/well). After an initial 24-h incubation period in a controlled incubator, the cells were exposed to various AIL concentrations (0.1, 0.3, 1, 3, 10, and 30 μmol/L) for an additional 24 h at 37°C under 5% CO_2_. Each AIL concentration was tested in four replicate wells. After exposure, 10 μL of a 5 mg/mL MTT solution was added to each well, and the cells were incubated for 2 h. Following supernatant removal, 100 μL of dimethyl sulfoxide was added to each well, agitated in the dark for 5 min, and absorbance readings were collected at 490 nm. Cell viability (%) was determined by dividing the absorbance value of the test group by that of the control group and multiplying by 100. The experimental procedure was replicated thrice, and concentrations of 0.1 and 0.3 μmol/L were selected for subsequent experiments.

### 2.13 Cell cycle distribution and apoptosis rate analysis through flow cytometry

In 6-well culture plates, SW620 and HCT116 CRC cells in their logarithmic growth phase were inoculated at a density of 5 × 10^5^ cells per well and cultured for 24 h in a controlled incubator. Two groups, the control (fresh medium) and AIL treatment (0.1 and 0.3 μmol/L) groups, were established. Following 24 h of incubation, the cells were washed with phosphate-buffered saline (PBS), subjected to digestion, collected, and then washed twice with cold PBS.

For cell cycle analysis, the cells were first fixed in 75% cold ethanol at 4°C for 12 h, followed by two washes with cold PBS. Subsequently, they were stained with 10 µL of propidium iodide (PI) dye in the dark for 15 min. Finally, they were analyzed through flow cytometry.

To assess apoptosis, the cells were incubated with 5 µL Annexin V/FITC for 5 min at room temperature in the absence of light. Subsequently, 10 µL PI dye with 400 µL PBS was added, and flow-through was immediately analyzed.

### 2.14 Transwell assay for cell invasion and migration

SW620 and HCT116 CRC cells in the logarithmic growth phase were adjusted to an appropriate density for invasion and migration assays. A 0.2 mL suspension of serum-free cells was introduced into the upper chamber of a Transwell chamber with or without matrix gel in 24-well plates (cell density: 4 × 10^4^ cells per well). The lower chamber contained 600 μL culture medium containing 30% fetal bovine serum. Two groups, control and AIL treatment (0.1 and 0.3 μmol/L), were established. Transwell chambers were incubated for 24 h, after which the liquid within and outside the Transwell chamber was removed, and the chambers were rinsed thrice with PBS. Subsequently, cells were fixed with 1 mL of 4% paraformaldehyde for 15 min at room temperature. After three washes with PBS buffer, the cells were stained with 1 mL 0.1% crystal violet solution for 10 min at room temperature. After another three washes with PBS, any cells that had not invaded or migrated in the upper chamber of the Transwell were gently removed using cotton swabs. The chambers were subsequently examined under a microscope, following which they were transferred to a new 24-well plate. In each well, 800 μL 33% glacial acetic acid was added, and the plates were shaken for 5 min to achieve decolorization. Cell invasion and migration were quantified by measuring absorbance at 570 nm using an enzyme-linked immunoassay detector.

### 2.15 Immunofluorescence

CRC cells (SW-620, HCT-116) in logarithmic growth phase were implanted in 24-well plates containing climbing tablets at a density of 2 × 10^4^ cells per well, cultured in 600 μL medium for 24 h, the supernatant was discarded, and the complete medium was replaced in the normal group. The drug group received complete medium containing AIL at 0.1 μmol/L and 0.3 μmol/L, respectively. 24 h after administration, the protein expression levels of PI3K, AKT and p-AKT were detected by immunofluorescence method. The detection methods are as follows: PBS wash for 3 min × 3 times, 4% paraformaldehyde fixed cells for 15 min, PBS wash for 3 min × 3 times; add TritonX100 to permeate for 10 min, dry and wash with PBS for 3 min × 3 times; 5% BSA occluded for 40 min without washing. Primary antibodies against specific proteins (PI3K, AKT, p-AKT, 1:200) were purchased from Abcam (MA, United States) and added to incubate overnight at 4°C, removed the next day and re-warmed for 1 h and washed with PBS for 5 min × 3 times. The cells were incubated with anti-light plus fluorescent secondary antibody (1:200) for 1 h at room temperature, washed with PBS for 5 min × 3 times, stained with DAPI for 8 min and washed for 5 min × 3 times, sealed with anti-fluorescence quencher, observed and photographed under a fluorescence microscope, and the results were calculated by optical density.

### 2.16 Statistical analysis

Statistical analysis was performed using SPSS version 26.0 software. Data are presented as means ± standard deviations (mean ± SD). Differences between groups were assed using either one-way analysis of variance or *t*-test, with *p* < 0.05 indicating statistical significance.

## 3 Results

### 3.1 Evaluation of ADME

This study assessed the pharmacodynamic activity of AIL by integrating Lipinski’s rule, oral bioavailability (OB), and drug-likeness (DL). Lipinski’s rule comprises several criteria, including molecular weight (MW < 500), lipid–water partition coefficient (−2 < AlogP <5), hydrogen bond donors (Hdon <5), hydrogen bond acceptor count (Hacc <10), and rotational bonds (RBN <10). DL indicates robust clinical efficacy; a higher DL value suggests greater drug-generating potential and OB is a critical pharmacokinetic parameter for orally administered drugs. DL and OB were considered key indicators of drug effectiveness. Using the Traditional Chinese Medicine Systems Pharmacology.

(TCMSP) database, the following criteria were employed for drug screening: OB = 20%, DL = 0.1, and RBN <10. According to our evaluation, the AIL parameters were as follows: OB = 27.96%, DL = 0.74, and RBN = 0. These results indicate highly promising pharmacodynamic activity (refer to Table 1 for detailed ADME parameters).

**TABLE 1:** ADME parameters for AIL.

Name	Hdon	Hacc	AlogP	RBN	OB (%)	Caco2	BBB	DL	TPSA (Å)
Ailanthone	3	7	−0.32	0	27.96	−0.59	−0.92	0.74	113.29^2^

### 3.2 RNA sequencing data and DEG analysis RNA

**FIGURE 1:**
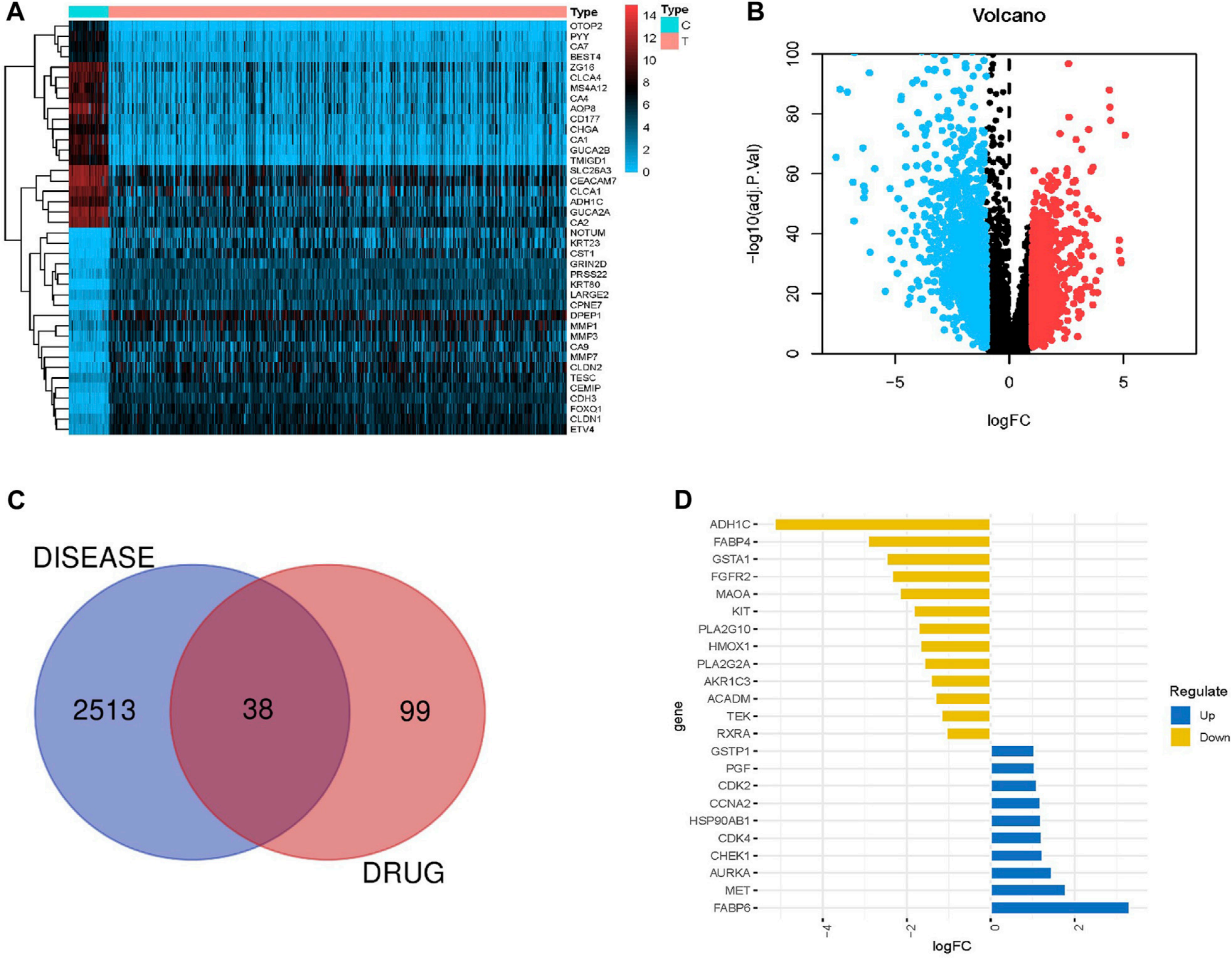
Differential expression of genes in CRC intersecting with AIL target genes. **(A,B)** Heatmaps and volcano plots showing differentially expressed genes in CRC (normal and tumor groups). Blue and red indicate downregulated and upregulated genes, respectively, whereas black indicates genes without differential expression. **(C)** Venn diagram showing 38 intersecting genes between the differentially expressed genes in CRC and AIL target genes. **(D)** Top 10 genes (upregulated and downregulated) among 38 intersecting genes.

By calculating gene expression differences between 41 normal samples and 446 CRC samples, we identified 2,551 differentially expressed genes (DEGs), including 1,125 upregulated genes and 1,426 downregulated genes (Supplementary Tables S1–S3). Figures 1A, B show the heatmap and volcano map illustrating DEGs, respectively.

### 3.3 Target analysis of AIL

Analysis of TCMSP, HERB, Swiss Target Prediction, and PharmMapper databases identified 137 target genes of AIL (Supplementary Table S4).

### 3.4 Intersection of disease genes and drug genes

Venn diagrams were obtained by intersecting DEGs in CRC with AIL target genes (Figure 1C). In total, 38 genes were enriched in both the groups (Supplementary Table S5). The top 10 upregulated genes included FABP6, MET, AURKA, CHEK1, CDK4, HSP90AB1, CCNA2, CDK2, PGF, and GSTP1 (Figure 1D). The top 10 downregulated genes included ADH1C, FABP4, GSTA1, FGFR2, MAOA, KIT, PLA2G10, HMOX1, PLA2G2A, and AKR1C3 (Figure 1D).

### 3.5 PPI network construction and network topology analysis

**FIGURE 2:**
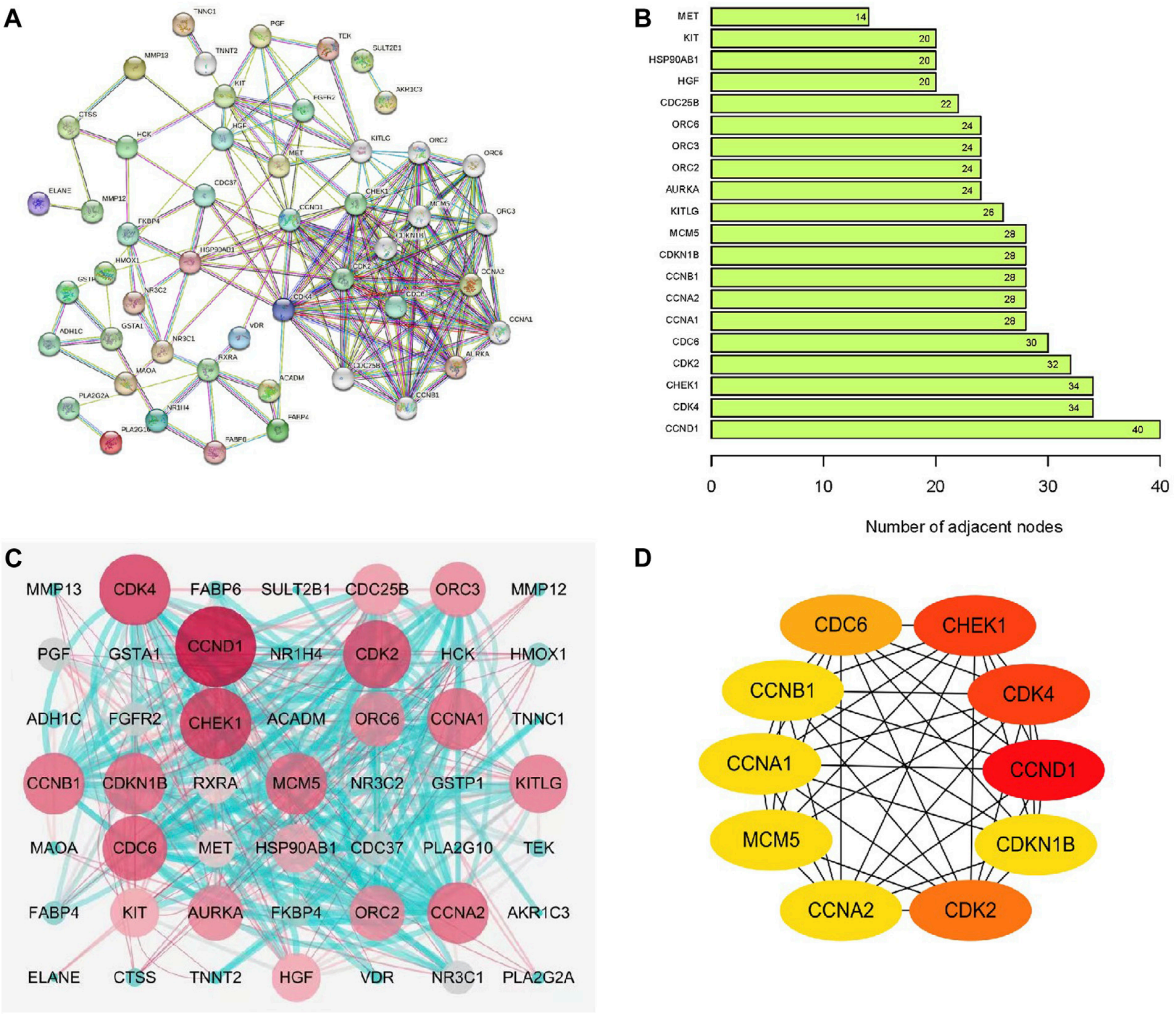
Construction of PPI network and network topology analysis. **(A)** STRING-based analysis of target protein interactions. **(B)** Histogram illustrating protein interactions associated with AIL. **(C)** Target protein interactions visualized using Cytoscape 7.2.1. **(D)** Top 10 differentially expressed genes determined via PPI screening.

Figure 2A presents the PPI analysis results of target proteins conducted using the STRING database. The top 20 differential genes within the network were arranged in a ranked manner (Figure 2B). A comprehensive analysis of DEGs derived from AIL-treated CRC was performed, following which these DEGs were evaluated and assigned scores using Cytoscape 7.2.1 software. A molecular network diagram was visualized using the “cytomubba” plugin (Figure 2C). For each target gene, the top 10 proteins with the highest number of neighboring genes were selected for statistical analysis (Figure 2D). Proteins with ≥30 connectivity included CDC6, CDK2, CHEK1, CDK4, and CCND1. Other proteins with high connectivity, including CDKN1B, CCNB1, CCNA1, MCM5, and CCNA2, were also obtained using PPI screening.

### 3.6 Verification of core genes

**FIGURE 3:**
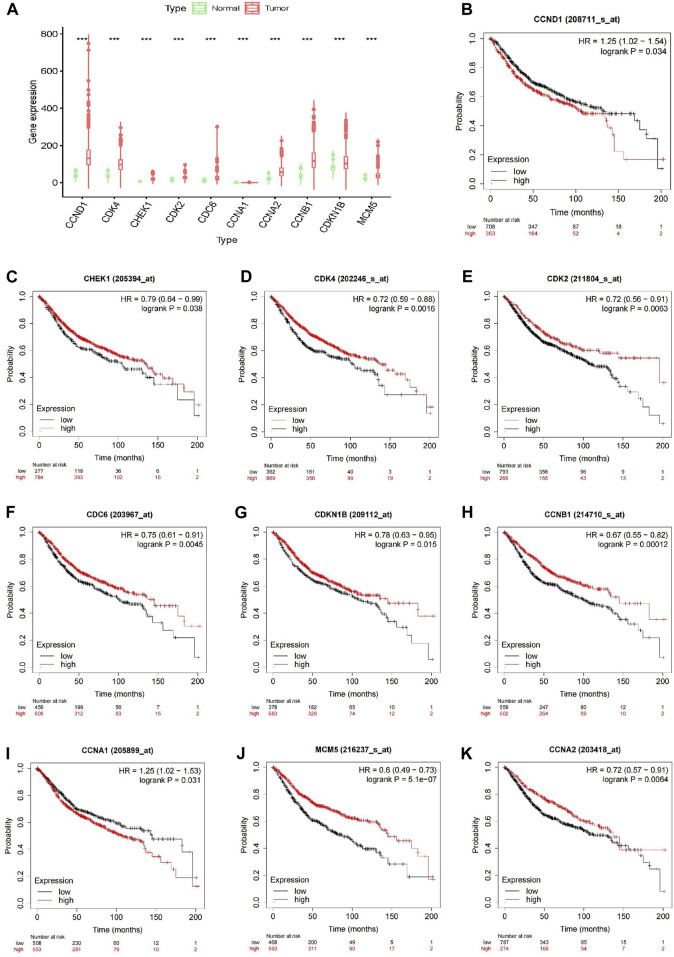
Validation of core genes. **(A)** Validation of differential expression of 10 core genes in normal and tumour tissues; **(B–K)** Validation of 10 core genes associated with survival in CRC patients by Kaplan-Meier analysis.

Expression analysis of the 10 core genes in the drug–target network, conducted in normal and tumor tissues, revealed significant differences (*p* < 0.001) in the expression levels of CCND1, CDK4, CHEK1, CDK2, CDC6, CCNA1, CCNA2, CCNB1, CDKN1B, and MCM5 (Figure 3A). In addition, the Kaplan-Meier online platform analysis indicated that the expression of these 10 core genes was significantly correlated with the survival of CRC patients (Figures 3B–K). Notably, a high expression of CCND1 and CCNA1 emerges as a prognostic indicator for unfavorable prognosis.

### 3.7 GO and KEGG enrichment analyses

**FIGURE 4:**
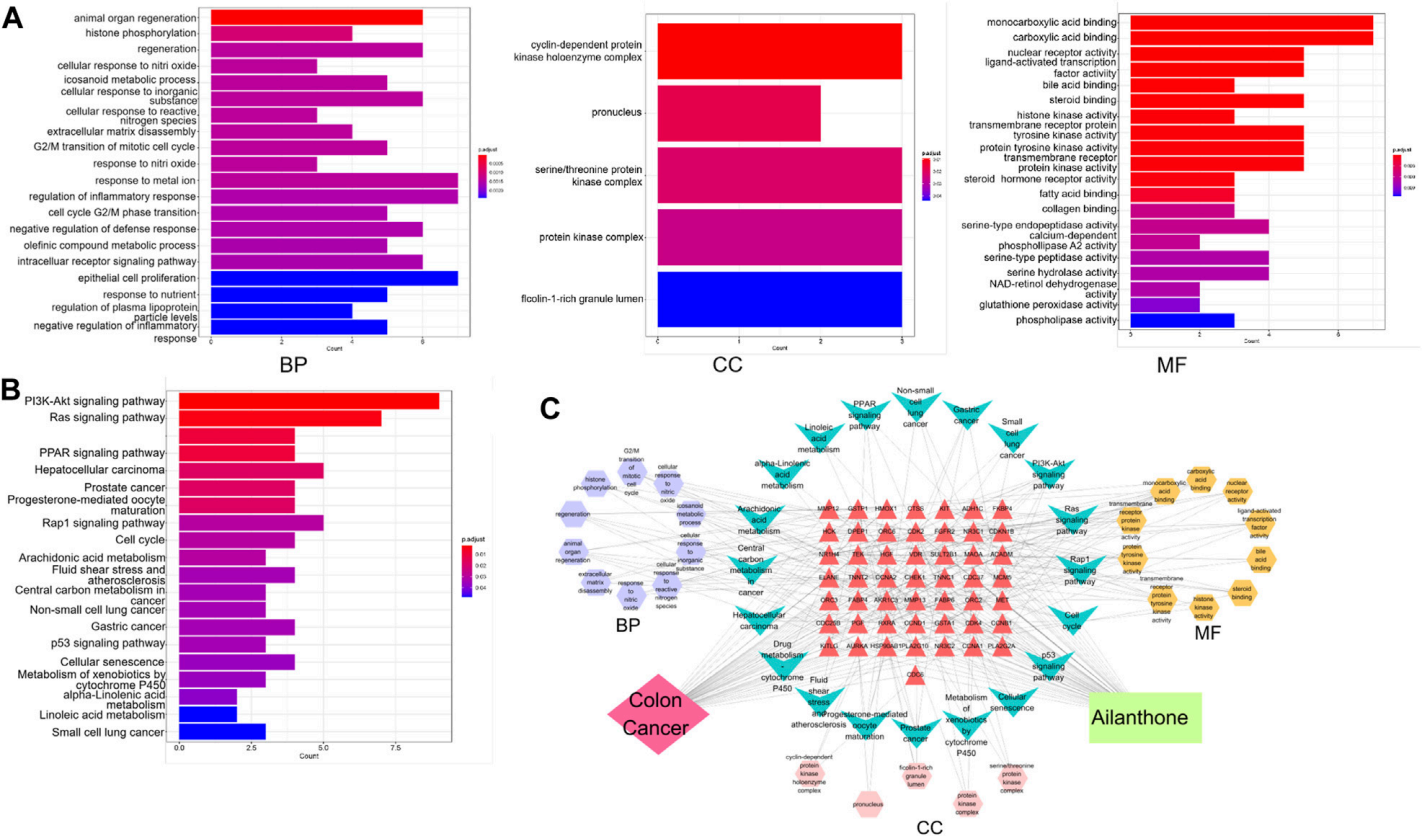
GO and KEGG Enrichment Analyses. **(A)** Enrichment analysis of DEGs, categorized into BP, CC, and MF terms. **(B)** KEGG pathway enrichment analysis of DEGs. **(C)** Diagram of the regulatory network denoted “AIL–CRC–target gene–KEGG.”

Enrichment analyses of GO gene functions and KEGG pathways were performed using the R programming language, and AIL targets in CRC were identified. Among the most enriched GO terms in Biological Process (BP) were animal organ regeneration, histone phosphorylation, regeneration, eicosanoid metabolic processes, and cellular responses to inorganic substances. The most enriched terms in Cellular Component (CC) and Molecular Function (MF) included the cyclin-dependent protein kinase holoenzyme complex and monocarboxylic acid binding, respectively (Figure 4A). In KEGG pathway analysis, the core genes in the network were primarily associated with cancer-related pathways, including the PI3K-AKT signaling pathway, Ras signaling pathway, drug metabolism (cytochrome P450), PPAR signaling pathway, and hepatocellular carcinoma (Figure 4B). Functional enrichment analysis results were visualized using Cytoscape 7.2.1. Thus, a regulatory network diagram termed as “AIL–CRC–Target Gene–KEGG” was established (Figure 4C), and the drug–disease relationships along with the pathways involved were analyzed.

### 3.8 Validation of molecular docking between AIL and targets

**FIGURE 5:**
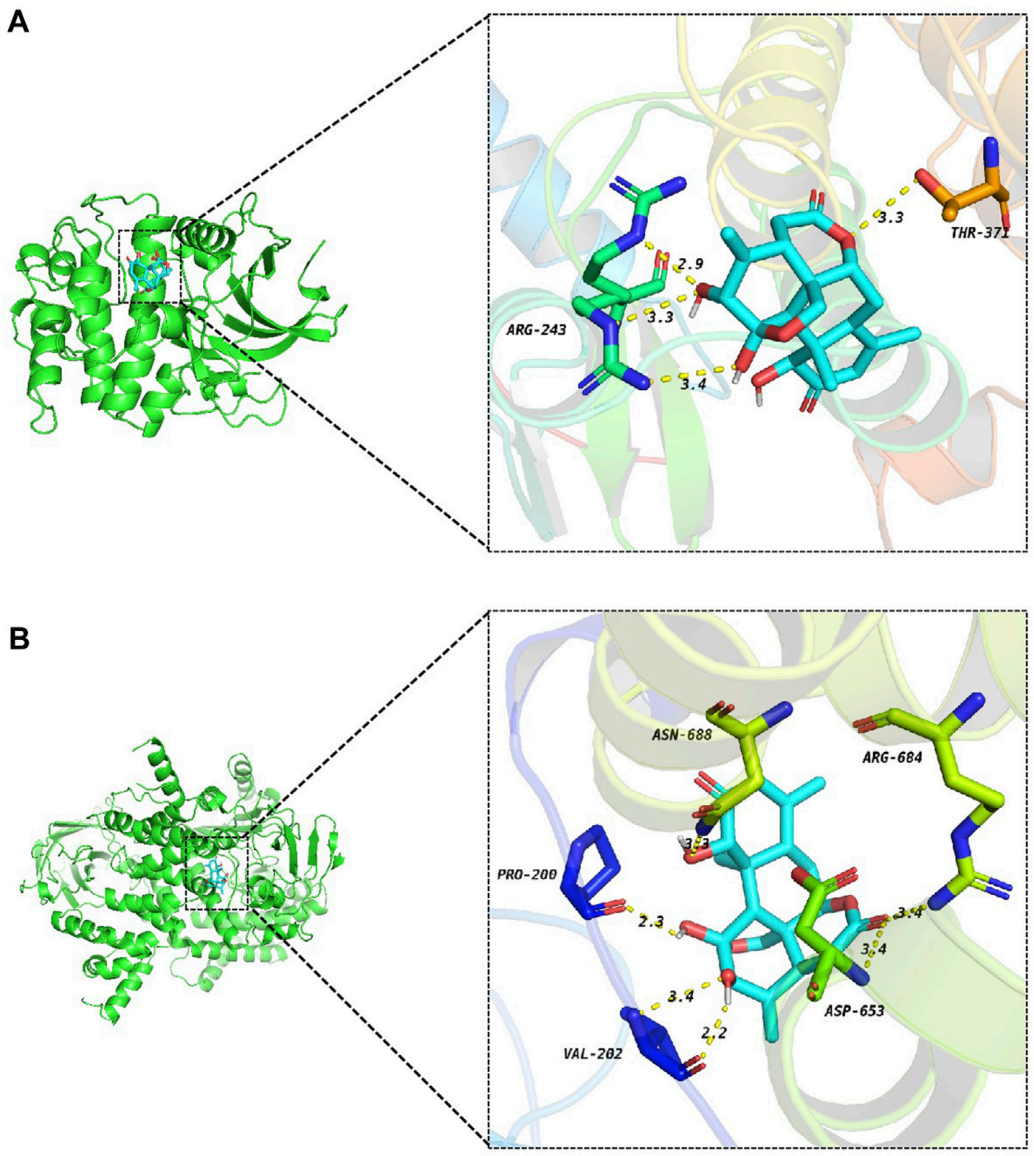
Docking diagram of AIL and target protein. **(A)** AIL-AKT, **(B)** AIL-PI3K.

The Protein Data Bank (PDB) was searched for protein crystal structures corresponding to the genes we need. It is generally believed that the binding energy between small molecules and proteins is ≤ −5.0 kJ/mol, meaning that both have good binding activity (Liao et al., 2023). Our docking results show that the binding energy of AIL to PI3K and AKT is the lowest, which is −8.6 kcal/mol and −7.5 kcal/mol, respectively (Supplementary Table S6), indicating that AIL has a strong binding affinity with PI3K and AKT. Hydrogen bonding plays a major role in the stability of compound-target binding (Iksen et al., 2023). As shown in Figure 5, AIL and AKT form three hydrogen bonds at ARG-243 and one hydrogen bond at THR-371. AIL and PI3K form two hydrogen bonds at VAL-202 and one hydrogen bond each at PRO-200, ASN-688, ARG-684, and ASP-653.

### 3.9 Effects of AIL on cell proliferation, apoptosis, and the cell cycle in CRC

**FIGURE 6:**
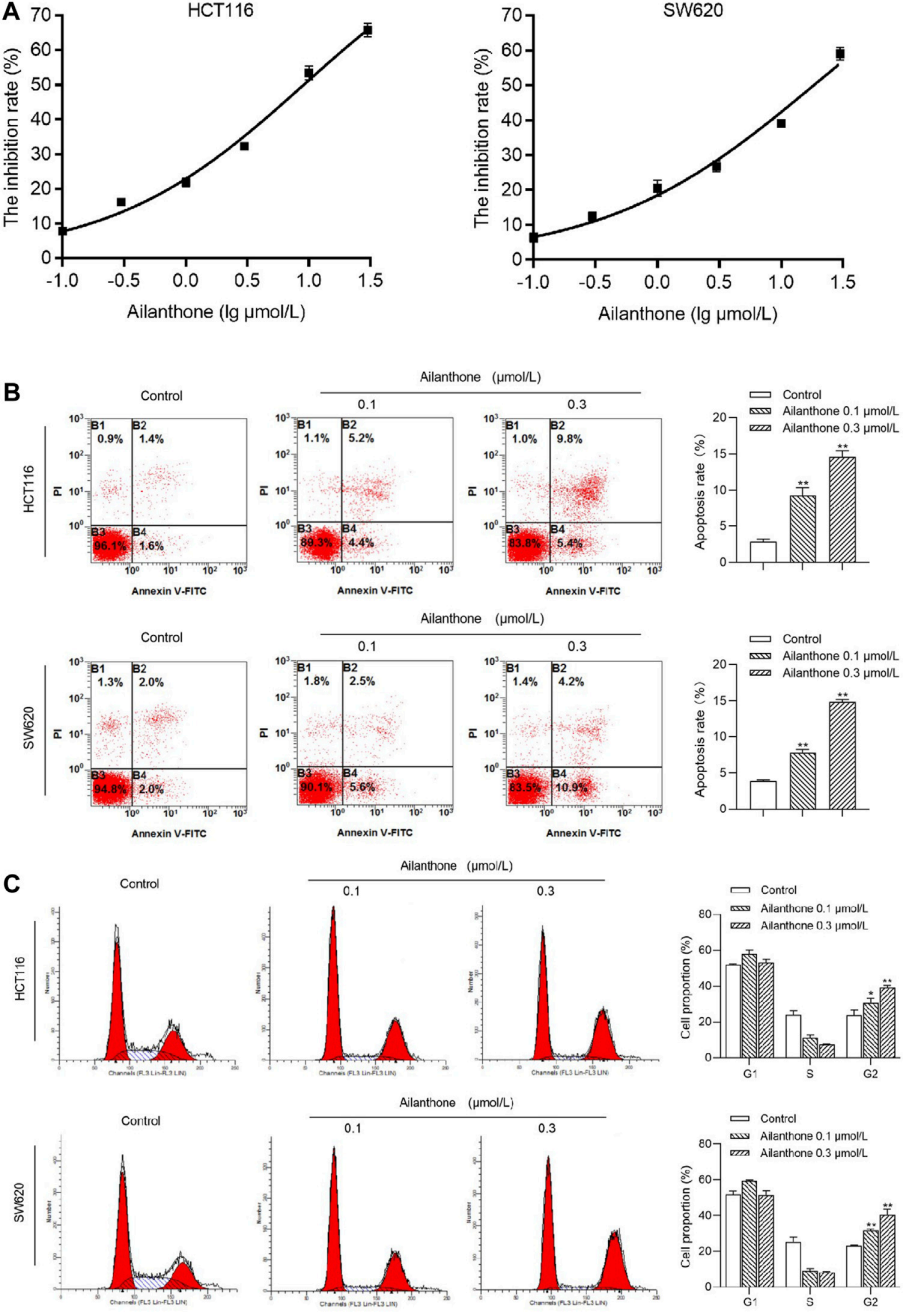
Effects of AIL on Proliferation, Apoptosis, and Cell Cycle in HCT116 and SW620 CRC Cells. **(A)** MTT assay outcomes of HCT116 and SW620 cell viability after treatment with 0–30 μmol/L AIL for 24 h. The IC50 values of AIL concentrations in HCT116 and SW620 CRC cells were 9.16 ± 0.93 and 18.42 ± 1.77 μmol/L, respectively. **(B)** Effects of AIL on HCT116 and SW620 CRC cell apoptosis. Increasing AIL concentrations significantly increased CRC cell apoptosis compared with the control group (*p* < 0.01). **(C)** Effects of AIL on the cell cycle of HCT116 and SW620 CRC cells. AIL treatments (0.1 and 0.3 μmol/L) notably reduced the number of CRC cells in the S phase but increased cell numbers in the G2 phase compared with the control cells (*p* < 0.05 and *p* < 0.01). Data are expressed as mean ± SD, n = 3. **p < 0.01 compared to control.

The effects of 24-h AIL treatment at varying concentrations (0.1, 0.3, 1, 3, 10, and 30 μmol/L) on SW620 and HCT116 CRC cell proliferation were assessed using the MTT assay. AIL exhibited a significant and dose-dependent inhibition of this proliferation in SW620 and HCT116, with half-maximal inhibitory concentration values of 9.16 ± 0.93 and 18.42 ± 1.77 μmol/L, respectively (Figure 6A). Notably, at 0.1 and 0.3 μmol/L AIL, the HCT116 cell inhibition rates were 7.8% and 16%, whereas SW620 cells exhibited inhibition rates of 6.4% and 12%, respectively; thus, subsequent investigations focused on the AIL concentrations of 0.1 and 0.3 μmol/L (Supplementary Table S7).

Flow cytometry analysis revealed a concentration-dependent increase in apoptosis in HCT116 and SW620 CRC cells following AIL treatment compared with that in the control group (Figure 6B). Treatment with AIL (0.1 and 0.3 μmol/L) significantly reduced HCT116 and SW620 CRC cells in the S phase coupled with a marked increase in cells in the G2 phase of the cell cycle compared with that in the control group (Figure 6C). This indicates that AIL effectively blocked the G2 phase in CRC cells, impacting cell proliferation.

### 3.10 Effect of AIL on CRC cell migration and invasion

**FIGURE 7:**
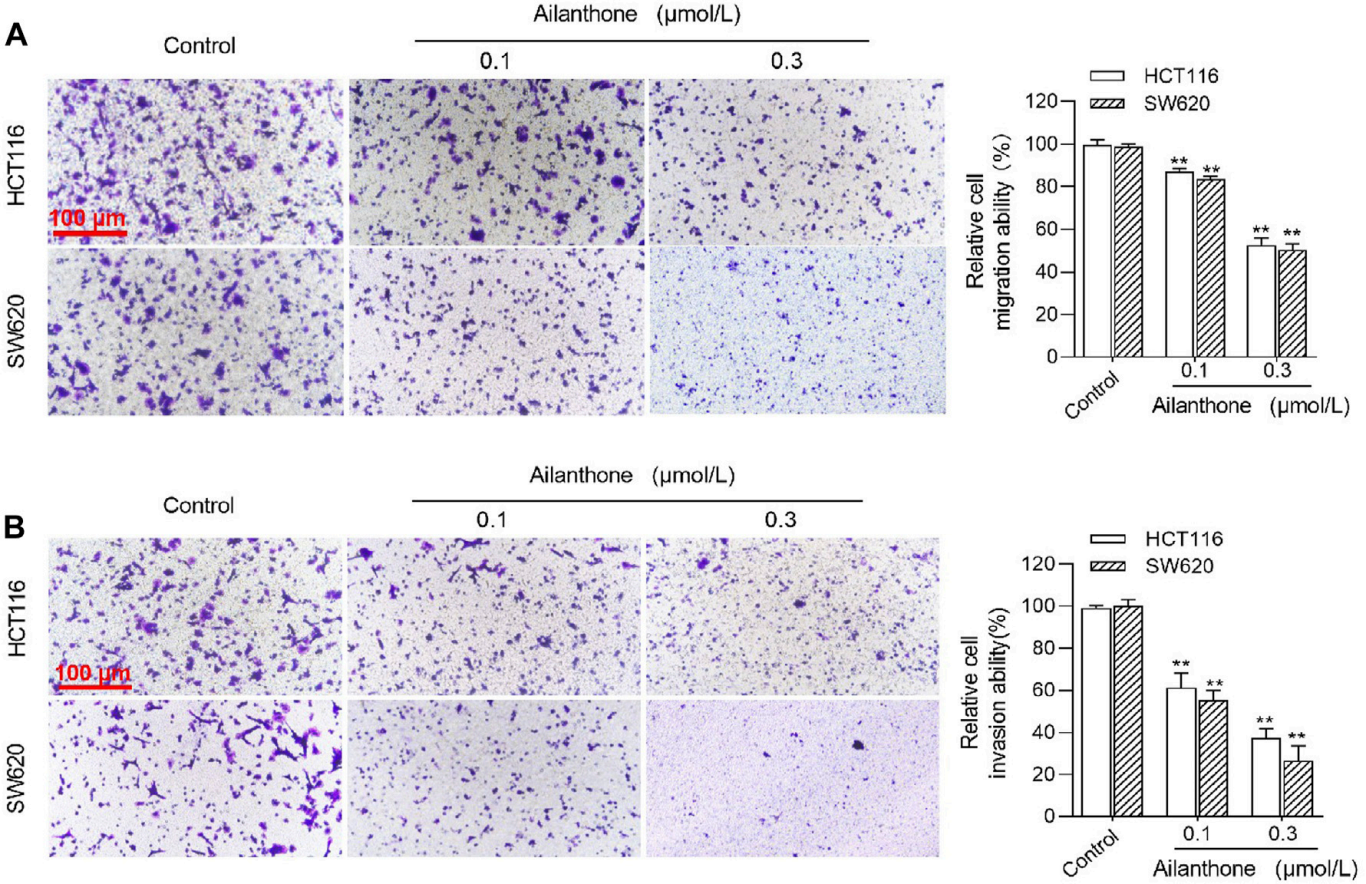
Effects of AIL on CRC Cell Migration and Invasion. The migration **(A)** and invasion **(B)** effects of AIL on HCT116 and SW620 CRC cells were examined using the Transwell assay, where AIL significantly inhibited both invasion and migration in a concentration-dependent manner compared with to control group. Data are expressed as mean ± SD, n = 3. **p < 0.01 compared to control.

Transwell chambers with or without matrix gel were used to assess the effect of AIL treatment (0.1 and 0.3 μmol/L) on the migration and invasion of SW620 and HCT116 CRC cells in the logarithmic growth phase. AIL exerted a concentration-dependent inhibitory effect on the migration and invasion abilities of both cell types compared to the control group (Figure 7).

### 3.11 Effect of AIL on PI3K/AKT signaling in CRC cells

**FIGURE 8:**
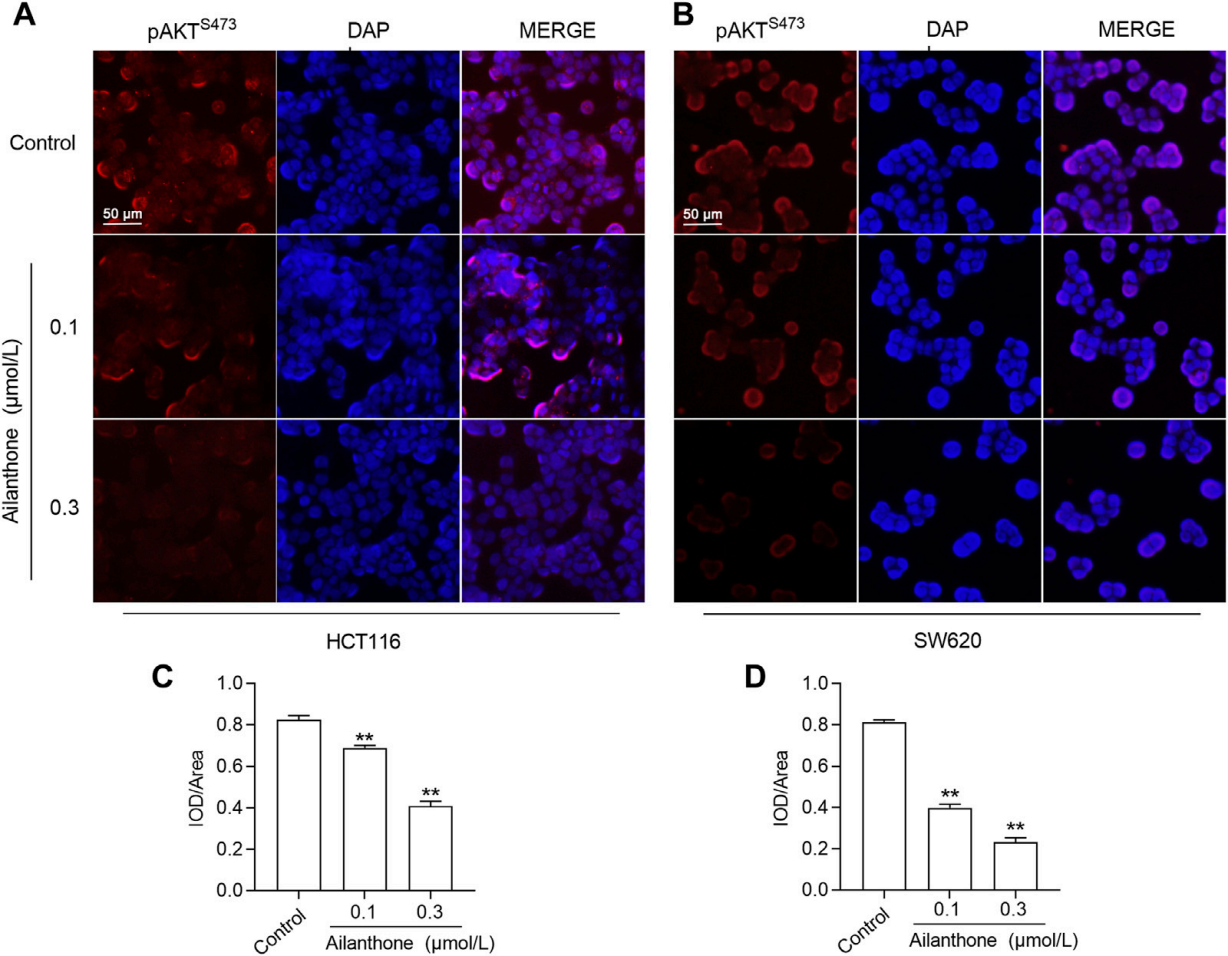
Immunofluorescence analysis of the effect of AIL on the PI3K/AKT pathway. **(A,B)** The expression of p-AKT^S473^ was detected by immunofluorescence. **(C,D)** The expression level of p-AKT^S473^ was calculated by the ratio of fluorescence intensity to area. Data are expressed as mean ± SD, n = 3. **p < 0.01 compared to control.

KEGG enrichment analysis results indicated that PI3K/AKT may be a key signaling pathway in the AIL-mediated treatment of CRC. The key to activation of the PI3K/AKT pathway is phosphorylation of the AKT protein, and phosphorylation of the AKT protein at S473 indicates activation of the PI3K/AKT pathway (Chen et al., 2022). Therefore, we detected the expression level of key proteins in the PI3K/AKT pathway in AIL-treated cells by immunofluorescence. The results showed that compared with the control group, the expression levels of PI3K and AKT protein in CRC cells treated with AIL did not show any significant change (Supplementary Figures S2, S3), but the expression level of p-AKT^S473^ protein was significantly decreased (*p* < 0.01), indicating that AIL could inhibit AKT phosphorylation, thereby inhibiting the activation of the PI3K/AKT signaling pathway and thus inhibiting the proliferation and metastasis of CRC cells, as shown in Figure 8.

## 4 Discussion

CRC constitutes a major health threat with high incidence and mortality rates (Hu et al., 2023). Although AIL reportedly exhibits promising antitumor activity (Zhuo et al., 2015; Ni et al., 2017; Yang et al., 2018; Liu et al., 2019), research regarding its role in CRC treatment is limited (Ding et al., 2022). To address this gap, we combined network pharmacology and bioinformatics to comprehensively assess the potential of AIL in CRC treatment. This innovative methodology not only highlighted the effects of AIL but established a theoretical foundation for future studies on these effects.

This study evaluated the drug-like properties of AIL using the SwissADME database by applying Lipinski’s rule, a guideline for drug design and screening (Liu et al., 2023). The results revealed that the ADME parameters of AIL (MW = 376.40 Da; Hdon = 3; Hacc = 7; AlogP = −0.32; RBN = 0) meet Lipinski’s rule parameters, suggesting satisfactory medicinal properties. Furthermore, applying the TCMSP database screening principles, AIL exhibited promising pharmacodynamic activity parameters (OB = 27.96%; DL = 0.74; RBN = 0). The analysis of integrated data from TCMSP, HERB, Swiss Target Prediction, and PharmMapper databases identified 137 target genes. RNA sequencing data from 487 samples revealed 2,551 DEGs in CRC, including 1,125 and 1,426 upregulated and downregulated genes, respectively. Intersecting CRC DEGs with AIL target genes yielded 38 intersecting genes.

To obtain a deeper understanding, PPI network analysis was used to identify the top 10 target genes for AIL in CRC, which included CCND1, CDK4, CHEK1, CDK2, CDC6, CDKN1B, CCNB1, CCNA1, MCM5, and CCNA2. Among these genes, CCND1, a key cell cycle regulator, is closely associated with CRC progression (Mustafi et al., 2012). Downregulating CCND1 inhibits CRC cell proliferation, migration, and invasion (Li et al., 2021c; Tu et al., 2022). CDK4 and CDK2, both members of the serine/threonine kinase family, primarily affect the G1 to S phase transition of the cell cycle (Lamb et al., 2003). Notably, in PI3K/AKT pathway–activated CRC cells, CDK2 and CDK4 upregulation reportedly promotes apoptosis (Hong et al., 2020), underscoring the intricate interactions between signaling pathways and cell cycle regulation in CRC. Furthermore, CHEK1 responds to DNA damage in CRC cells and TP53 status influences its function (Gali-Muhtasib et al., 2008). CDC6 is overexpressed in CRC tissues and has been correlated with metastasis and prognosis (Yang et al., 2022). Thus, CDC6 may serve as a valuable biomarker and a potential therapeutic target for CRC. Moreover, CDKN1B, initially associated with physical activity in patients with CRC, has been linked to metastasis (Morikawa et al., 2011; Pavlic et al., 2021). CCNB1, a key regulator of cell proliferation and G2/M phase progression (Yuan et al., 2021), forms a complex with CDK1, namely, the cyclin B1/CDK1 complex, which is essential for G2/M phase transition and mitosis initiation (Fang et al., 2019; Zhang et al., 2022). Importantly, G2/M phase transition and CDK1 expression are influenced by PI3K/AKT signaling (Wang et al., 2017). CCNB1 shRNA arrests CRC cells in the G2/M phase, thereby inhibiting their growth (Fang et al., 2014). The inhibition of CCNB1 expression has also been reported to inactivate PI3K/AKT signaling and induce G2/M arrest (Meng et al., 2023). Moreover, elevated CCNA1 and CCNA2 expression levels activates the PI3K/AKT pathway, promoting tumor growth and cell survival (Talaat et al., 2022). MCM5 is a chromatin-binding protein that serves as a diagnostic marker for CRC, regulating tumor cell proliferation and cell cycle progression (Dai et al., 2017; Huang et al., 2018). Collectively, these findings suggest that AIL exerts anti–CRC effects through multiple targets.

For a comprehensive exploration of interacting gene functions, GO and KEGG analyses were performed. Interacting genes were primarily involved in the BPs such as animal organ regeneration, histone phosphorylation, regeneration, eicosanoid metabolism, and cellular responses to inorganic substances. Histone phosphorylation correlates strongly with colorectal cancer progression, playing a pivotal role in cell mitosis (Okugawa et al., 2015). In addition, the PI3K/AKT pathway modulates proto-oncogene expression by inducing histone phosphorylation (Ibuki et al., 2014). KEGG pathway analysis and the BP-related interactive genes further supported this finding, indicating participation in the PI3K-AKT, Ras, metabolism (cytochrome P450), PPAR, and hepatocellular carcinoma signaling pathways. Abnormal PI3K/AKT signaling plays a central role in CRC initiation and development, influencing its progression and prognosis (Bishnupuri et al., 2019). Additionally, we performed molecular docking analysis between AIL and potential core targets identified by PPI analysis and key proteins PI3K and AKT in the PI3K/AKT signaling pathway screened by KEGG enrichment analysis. The docking results showed that AIL had the strongest binding affinity with PI3K and AKT (AIL had the lowest binding energy with PI3K and AKT of −8.6 kcal/mol and −7.5 kcal/mol, respectively) (Ren et al., 2023), indicating that PI3K and AKT are key targets for the anti-cancer activity of AIL. Taken together, these findings suggest that the anti–CRC effects of AIL involve multiple signaling pathways, with the PI3K/AKT pathway emerging as a central player. Notably, the PI3K/AKT pathway differs from the regulatory mechanism of AIL in other tumors mentioned above, such as non-small cell lung cancer (Ni et al., 2017), acute myeloid leukemia (Wei et al., 2018), bladder cancer (Daga et al., 2019), breast cancer (Wang et al., 2018), gastric cancer (Chen et al., 2017), etc.

The results of apoptosis and cell cycle assays performed in this study support the above findings and demonstrate that AIL induces apoptosis and arrests CRC cells in G2 phase, thereby inhibiting proliferation, consistent with previous findings (Su et al., 2006; Yang et al., 2020; Yi et al., 2021). The PI3K/AKT pathway plays a pivotal role in the onset and progression of colorectal cancer, influencing autophagy, apoptosis, and EMT-mediated cancer cell migration and invasion, accompanied by regulation of CCND1, mTOR, FOXO, HIF-1α, CASP, Bax, and Bcl-2 expression (Wei et al., 2019; Xia et al., 2021; Yu et al., 2022; Chen et al., 2023). Our immunofluorescence results showed that AIL significantly inhibited the phosphorylation of AKT protein at the S473 site and suppressed the activation of the PI3K/AKT signaling pathway, thereby inhibiting the proliferation and metastasis of CRC cells.

This study introduces a novel approach for evaluating the potential of AIL in CRC therapy. AIL exhibits promising drug-like properties and pharmacodynamic activity, thereby rendering it a potential candidate for further investigation in CRC treatment. Through the comprehensive analysis of target genes and pathways, the potential mechanisms underlying the anti–CRC effects of AIL were explored. The PI3K/AKT pathway emerged as a central player and may be a key target for future interventions in CRC treatment and prognosis. These findings provide valuable insights into the multifaceted effects of AIL on CRC, offering a foundation for future research and potential therapeutic applications.

However, it is important to note that this study serves as a preliminary exploratory investigation, and the evidence presented indicates the potential of AIL in the treatment of CRC. Furthermore, it is imperative to conduct additional *in vivo* experiments to gain a comprehensive understanding of the precise relationships and mechanisms underlying AIL’s effects on key targets and signaling pathways, such as PI3K/AKT.

## 5 Conclusion

AIL exerts antitumor effects in CRC through multiple targets and pathways. A pivotal mechanism is that AIL inhibits the activation of the PI3K/AKT pathway, which arrests the cell cycle in G2, induces apoptosis and prevents tumour metastasis.

## Data Availability

The original contributions presented in the study are included in the article/Supplementary Material, further inquiries can be directed to the corresponding authors.
