# Supplementary figures and images for: Elucidation of the mechanism of action of ailanthone in the treatment of colorectal cancer: integration of network pharmacology, bioinformatics analysis and experimental validation
Source: Front Pharmacol. 2024 Feb 7;15:1355644. doi: 10.3389/fphar.2024.1355644 (PMC10880095; doi:10.3389/fphar.2024.1355644)

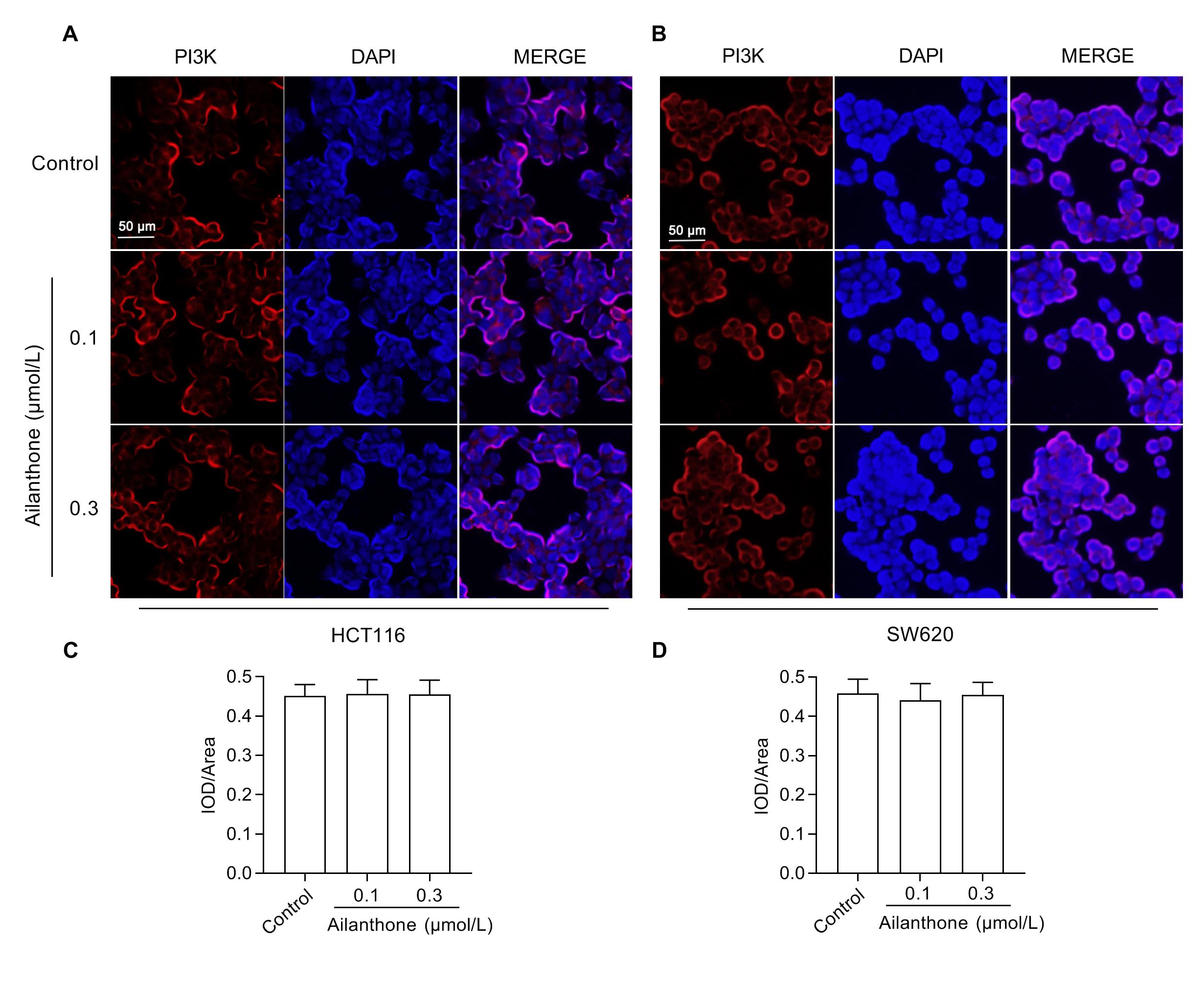

Supplement: Supplementary file 1 [file DataSheet1.ZIP › Supplementary figures/Supplementary Fig. 2.jpg]

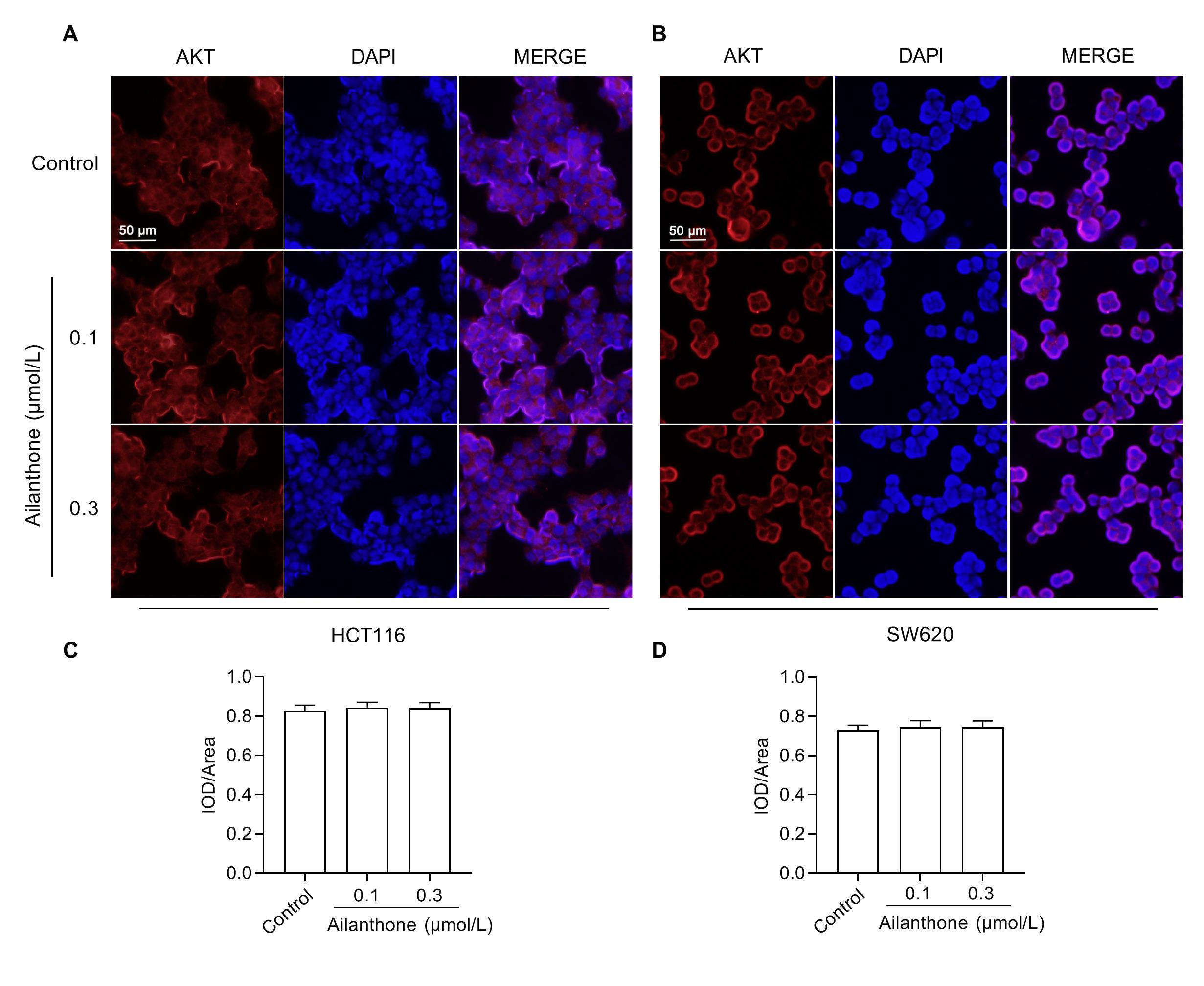

Supplement: Supplementary file 1 [file DataSheet1.ZIP › Supplementary figures/Supplementary Fig. 3.jpg]
