# Supplementary material for: Elucidation of the mechanism of action of ailanthone in the treatment of colorectal cancer: integration of network pharmacology, bioinformatics analysis and experimental validation
Source: Front Pharmacol. 2024 Feb 7;15:1355644. doi: 10.3389/fphar.2024.1355644 (PMC10880095; doi:10.3389/fphar.2024.1355644)
Supplement: Supplementary file 2 [file DataSheet2.ZIP › Supplementary tables/supplementary table 7.doc]

Supplementary Table 7-1.Effects of 0.1, 0.3, 1, 3, 10, and 30 μmol/L Ailanthone on colon cancer cells HCT116

| Concentration (μmol/L) | Inhibition rate (%) |
| --- | --- |
| 0.1 | 7.84±0.67 |
| 0.3 | 16.16±0.51 |
| 1 | 21.85±1.25 |
| 3 | 32.25±0.87 |
| 10 | 53.39±2.06 |
| 30 | 65.79±1.92 |

Supplementary Table 7-2. Effects of 0.1, 0.3, 1, 3, 10, and 30 μmol/L Ailanthone on colon cancer cells SW620

| Concentration (μmol/L) | Inhibition rate (%) |
| --- | --- |
| 0.1 | 6.37±1.32 |
| 0.3 | 12.42±1.23 |
| 1 | 20.49±2.34 |
| 3 | 26.70±1.50 |
| 10 | 39.12±0.78 |
| 30 | 59.02±1.75 |
